# Multi-feature fusion-based consumer perceived risk prediction and its interpretability study

**DOI:** 10.1371/journal.pone.0316277

**Published:** 2025-01-03

**Authors:** Lin Qi, Yunjie Xie, Qianqian Zhang, Jian Zhang, Yanhong Ma

**Affiliations:** 1 School of Economics & Management, Beijing Information Science & Technology University, Beijing, China; 2 Beijing Key Lab of Green Development Decision Based on Big Data, Beijing, China; 3 Beijing World Urban Circular Economy System (Industry) Collaborative Innovation Center, Beijing, China; Ataturk University, TÜRKIYE

## Abstract

E-commerce faces challenges such as content homogenization and high perceived risk among users. This paper aims to predict perceived risk in different contexts by analyzing review content and website information. Based on a dataset containing 262,752 online reviews, we employ the KeyBERT-TextCNN model to extract thematic features from the review content. Subsequently, we combine these thematic features with product and merchant characteristics. Using the PCA-K-medoids-XGBoost algorithm, we developed a predictive model for perceived risk. In the feature extraction phase, we identified 11 key features that influence perceived risk in online shopping. During the prediction phase, the model performs excellently across different sample types in the test set, achieving a precision (P) of 84%, a recall (R) of 86%, and an F1 score of 85%. Through the model’s interpretability analysis, we find that quality, functionality, and price are key features affecting perceived risk for electronic products. In the case of skincare products, skin safety is the most critical feature. Additionally, there are significant differences in feature characteristics between high-risk samples and normal samples.

## 1 Introduction

With the rapid development of Internet technology and the increasing prevalence of e-commerce, online shopping has become an indispensable part of people’s lives. Statistics indicate that global retail e-commerce sales are projected to exceed 6.3 trillion USD by 2024 [1]. While consumers benefit from the convenience of online shopping, they simultaneously face perceived risks stemming from various uncertainties inherent in the e-commerce environment [2]. Excessively high perceived risk not only affects consumers’ purchasing decisions but may also lead to customer attrition, thereby impeding the sustainable development of e-commerce [3].

The perceived risk in online shopping stems from the information asymmetry between offline and online shopping experiences [4]. Unlike traditional offline shopping, consumers in e-commerce environments cannot directly interact with or experience products, making it challenging to comprehensively understand product quality and performance. Furthermore, the recurrence of issues such as online fraud and privacy breaches exacerbates consumers’ perception of uncertainty, leading to an increase in perceived risk [5]. Moreover, the overwhelming volume of product information and complex promotional strategies on e-commerce platforms often lead to confusion and anxiety among consumers during their decision-making process [6]. Consequently, the development of innovative measurement models using big data analytics and machine learning techniques to achieve a comprehensive quantitative assessment of perceived risk has become an urgent issue in e-commerce research.

However, the subjective, multidimensional, and dynamic nature of perceived risk poses significant challenges for accurate prediction in the context of online shopping environments [7]. In recent years, machine learning has gained widespread application in the domain of risk prediction. Through the analysis and learning of massive datasets, machine learning algorithms have demonstrated the capability to automatically identify risk patterns, enabling quantitative risk assessment and early warning mechanisms [8]. However, extant research has predominantly focused on financial domains such as financial risk and credit risk, with relatively limited exploration in the realm of perceived risk prediction [9]. Indeed, the vast repositories of user behavior data and online review content accumulated by e-commerce platforms encapsulate rich consumer perception information. Leveraging machine learning techniques to conduct in-depth mining of these datasets holds promise for achieving precise prediction of perceived risks in online shopping contexts [10]. Furthermore, prior research has employed techniques such as topic modeling and deep learning to analyze online review data, aiming to uncover the dimensions of consumer risk perception across diverse contextual scenarios [11, 12].

Considering the multidimensionality and timeliness of perceived risk, as well as the limited predictive capability of traditional surveys in e-commerce scenarios, this study proposes a comprehensive machine learning approach to predict perceived risk. This method not only effectively combines objective factors and subjective reviews but also addresses the interpretability challenges in practical predictive applications.

## 2 Literature review

### 2.1 Dimensional categorization of perceived risk in online shopping

According to Cox, the fundamental assumption underlying perceived risk theory is that consumer behavior is goal-oriented. Perceived risk arises when consumers are subjectively uncertain about which consumption choice (product, brand, etc.) will best satisfy their objectives [13]. Cox posited that perceived risk originates from multiple factors, a perspective that catalyzed academic exploration into the dimensions of perceived risk. Building on Cox’s research, Kaplan proposed a five-dimensional model of perceived risk, encompassing financial risk, performance risk, physical risk, psychological risk, and social risk [14]. Mitchell notes that existing literature mainly categorises perceived risks from the perspectives of physical risk, financial risk, functional risk, and social risk, which lacks consistency. He believes that the dimensions of perceived risk should be dynamically adjusted based on factors such as product type and purchasing context [15].

Compared to traditional brick-and-mortar shopping, online purchasing is characterized by information asymmetry and non-face-to-face transactions [16–18]. Crespo et al. developed an extended e-commerce acceptance model and proposed six dimensions of perceived risk in online shopping: financial, social, time, psychological, and privacy risks. Through a comparative analysis of two sample groups, they discovered that the financial dimension exhibited higher significance [19]. Kamalul employed quantitative analysis to test hypotheses through an online survey of 350 respondents. The findings revealed that financial risk, product risk, and security risk exerted significant negative influences on consumers’ online purchase intentions. In contrast, social risk was found to have no significant impact [20]. Based on the consideration of the emotional dynamics of online shopping, Zhang has introduced the importance-Kano model into the fresh produce e-commerce sector, proposing new dimensions such as product quality, delivery service, customer service, and discrepancies in descriptions [21]. These new dimensions reflect the uniqueness and complexity of perceived risk in the context of online shopping. The dimensions of perceived risk are summarized in Table 1.

**Table 1:** Summary of perceived risk dimensions.

Author(s) & Year	Dimensions of Perceived Risk
Kaplan (1974)	Financial Risk, Performance Risk, Physical Risk, Psychological Risk, Social Risk
Mitchell (1999)	Physical Risk, Financial Risk, Functional Risk, Social Risk, Product Type, Purchase Situation
Crespo (2009)	Financial, Social, Time, Psychological, Privacy
Kamalul (2018)	Financial, Product, Time, Safety, Social
Zhang (2023)	Product Quality, Delivery Service, Customer Service, Description Mismatch

### 2.2 Factors influencing perceived risk

In the context of online shopping, the factors influencing consumers’ perceived risk are characterized by diversity and complexity. These factors encompass traditional commercial elements such as product attributes and vendor characteristics, as well as e-commerce-specific elements like online reviews.

At the product level, Zheng et al. employed an ordered logit model to examine the frequency of consumers’ online food purchases. Their study considered various factors, including product attributes and consumer perceptions. The results revealed that product attributes exerted the most significant influence on perceived risk [22]. Chen et al. utilized a panel data regression model to analyze a large-scale dataset of product reviews from Amazon. Their findings indicated that product type is a crucial factor influencing customers’ perceived risk [23]. Wu integrated perceived risk theory, signaling theory, and equity theory to examine the impact of price variations on perceived risk. By manipulating product prices, Wu found that price dispersion positively influences perceived risk. Specifically, larger price differences were associated with higher levels of perceived risk among consumers [24].

At the merchant level, Hong integrated trust theory with the Technology Acceptance Model (TAM) to examine the antecedents of trust, including perceived risk and information quality. The study revealed an inverse relationship between vendor reputation and consumers’ perceived risk: the better the overall reputation of the vendor, the lower the perceived risk among consumers [25]. Chopdar combines signal theory with the stimulus-organism-response (S-O-R) framework and finds through a questionnaire survey that higher merchant transparency correlates with lower consumer perceived risk. This transparency is reflected in merchants’ proactive disclosure of information such as their background and qualifications [26]. Matute et al. investigated the mediating role of trust in the relationship between electronic word-of-mouth (eWOM) and purchase intention. Their findings revealed that enhancing the marketing effectiveness of eWOM can significantly reduce consumers’ perceived risk [27, 28].

At the level of online reviews, Roy et al. demonstrated that the information embedded within reviews exerts a significant influence on perceived risk [29]. Yadav et al. employed the Stimulus-Organism-Response framework to empirically examine the mediating role of perceived risk between online reviews and behavioral intentions. Their findings revealed that online reviews serve as a crucial reference for consumers in making purchase decisions and can effectively mitigate consumers’ perceived risk [30]. Furthermore, Moliner et al. demonstrated that in the context of online shopping, online reviews and word-of-mouth (WOM) emerge as critical factors influencing perceived risk. Their research indicated that positive reviews and WOM can effectively reduce consumers’ perceived risk [31]. To gain a more profound understanding of the impact of review characteristics on perceived risk, Yang et al. integrated Signaling Theory and the Heuristic-Systematic Model (HSM) to analyze various dimensions of reviews, including quantity, valence, and depth. Their findings indicated that a large number of positive and detailed reviews can effectively mitigate perceived risk [32].

In summary, the factors influencing perceived risk in online shopping exhibit complex characteristics across multiple levels and pathways. Factors such as insufficient product information, lack of merchant transparency, and an abundance of negative reviews tend to exacerbate risk perception. The factors influencing perceived risk are summarized in Table 2.

**Table 2:** Synthesis of research on factors influencing perceived risk in consumer behavior.

Influencing Factor	Author(s) & Year	Theoretical Framework
Product Factors	Zheng (2020)	Technology Acceptance Model (TAM), Theory of Planned Behavior (TPB), Trust Theory
	Chen (2021)	Consumer Utility Theory, Information Economics, Behavioral Decision Theory
	Wu (2015)	Signaling Theory, Perceived Risk Theory
Merchant Factors	Hong (2013)	Trust Theory, TAM, TPB
	Chopdar (2024)	Signaling Theory, Stimulus-Organism-Response
	Matute (2016)	TAM, Trust Theory
Online Review	Roy (2024)	Perceived Risk Theory, Mindfulness Theory
	Yadav (2024)	Perceived Risk Theory, TPB
	Yang (2016)	Perceived Risk Theory, Signaling Theory

### 2.3 Measurement of perceived risk

Methods for measuring perceived risk can be categorized into traditional approaches and machine learning based techniques. Traditional methods primarily employ a two-factor model, collecting respondent data through designed scales, questionnaire surveys, or interviews. These methods calculate perceived risk values by computing the two-dimensional relationship between risk dimensions and uncertainty [33]. In the context of e-commerce, the majority of scholars employ multi-item scales to measure consumers’ psychological perception dimensions. The design of these scales primarily draws upon existing literature while adapting to current research objectives. Typically, Likert scales are utilized for measurement [34, 35]. For instance, Bashir et al. developed a perceived risk scale for online shoppers comprising eight risk dimensions and 26 items. The scale underwent rigorous reliability and validity testing, demonstrating high internal consistency and robust construct validity. Results indicated that the scale effectively measures the perceived risk levels of online shoppers [36].

With the advancement of big data and artificial intelligence technologies, the methodologies for measuring perceived risk are evolving from traditional questionnaire-based surveys towards machine learning-driven multivariate data analysis. Lee et al. developed and validated a supervised machine learning model, GSVM (Generalized Support Vector Machine), utilizing physiological data collected from construction workers via wearable devices. The model achieved a prediction accuracy of 81.2% in distinguishing between low and high perceived risk levels, effectively addressing the subjectivity-objectivity issue in perceived risk measurement [37]. Trivedi et al. proposed an enhanced machine learning model based on stacked renowned classifiers, employing feature selection techniques to identify the most significant predictors. Through the analysis of consumers’ online shopping behavior data, they discovered that concerns related to privacy, security, and product quality, among others, contribute to increased perceived risk [38]. Rausch et al. employed various machine learning algorithms to predict online shopping cart abandonment behavior. Their findings indicate that higher levels of financial and time risks significantly increase the likelihood of consumers abandoning their shopping carts. Moreover, the authors discovered that most tree-based methods demonstrate superior predictive power compared to other machine learning approaches [39]. Furthermore, researchers have explored extracting perceived risk information from the vast corpus of consumer generated reviews on social media platforms. By applying text mining techniques to analyze this extensive user-generated content, they have effectively complemented traditional questionnaire-based surveys, enabling more precise measurement of perceived risk [40, 41]. Lin et al. leveraged social media review data and applied the BERT pre-trained language model to learn distributed representations of review texts. This approach enabled precise multi-dimensional prediction and sentiment analysis of perceived risk [42].

From the existing studies, it can be found that online reviews have an important impact on the prediction effect, but the current perceived risk feature mining is insufficient, and the existing studies fail to consider the characteristics of fragmented and spoken review texts, which leads to the low applicability of the traditional measurement models. Secondly, although machine learning models can adaptively acquire features and have been strengthened in prediction performance, the black box models constructed are still lacking in the interpretability of prediction effects.

## 3 Research methodology

**Fig 1:**
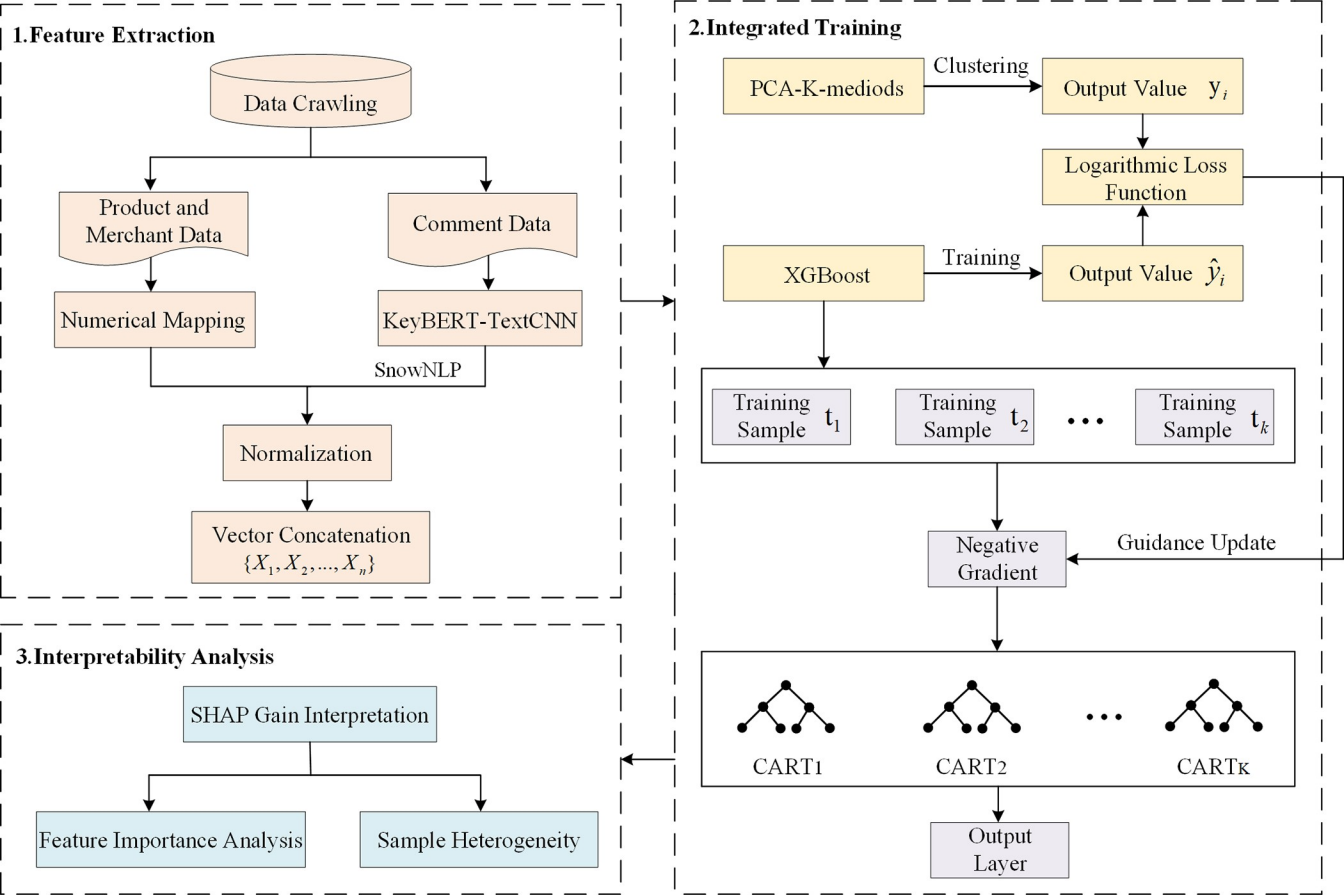
Research framework.

The model mainly includes three stages: feature extraction, ensemble training, and interpretability analysis. (1) Feature extraction phase: Perform numerical mapping of product and merchant data. Use KeyBERT-TextCNN to achieve keyword extraction and topic classification tasks for review texts. (2) Integrated training phase: Use the PCA-K-medoids clustering algorithm to generate risk category labels for the samples. Subsequently, train the XGBoost model for risk prediction. (3) Interpretability Analysis Phase: The interpretability analysis is completed by embedding SHAP values into the output layer of XGBoost. The detailed research framework is illustrated in Fig 1.

### 3.1 Risk feature extraction based on text topic analysis

#### 3.1.1 Topic classification based on KeyBERT-TextCNN

**Fig 2:**
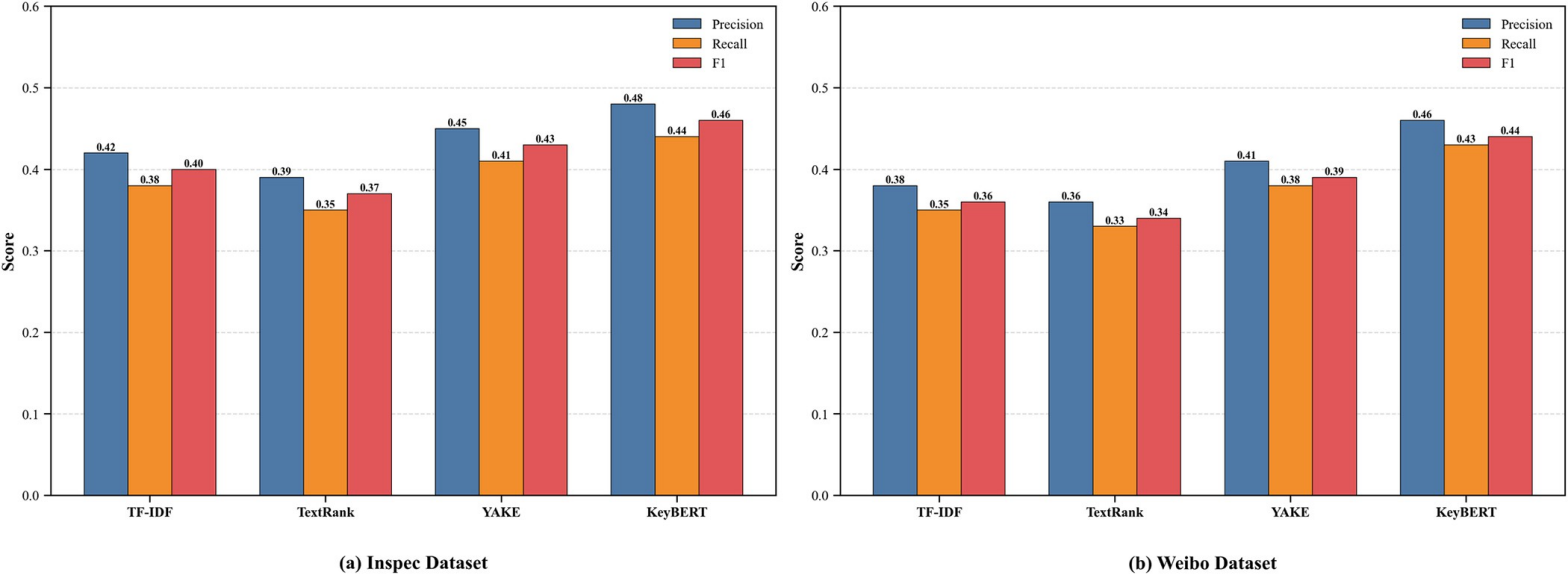
Comparison of keyword extraction effects. Extract the top-5 keywords (N = 5) from the keyword sequences output by each algorithm to form the benchmark parameter set.

In recent years, the BERT model, based on the Transformer architecture, has achieved remarkable success in various natural language processing (NLP) tasks. KeyBERT combines an attention mechanism to identify keywords that are highly relevant and informative to the document’s theme by calculating the semantic similarity between candidate keywords and the document [43]. As shown in Fig 2, KeyBERT maintains stable high performance when handling different languages and types of text.

#### 3.1.2 Text topic sentiment analysis

**Fig 3:**
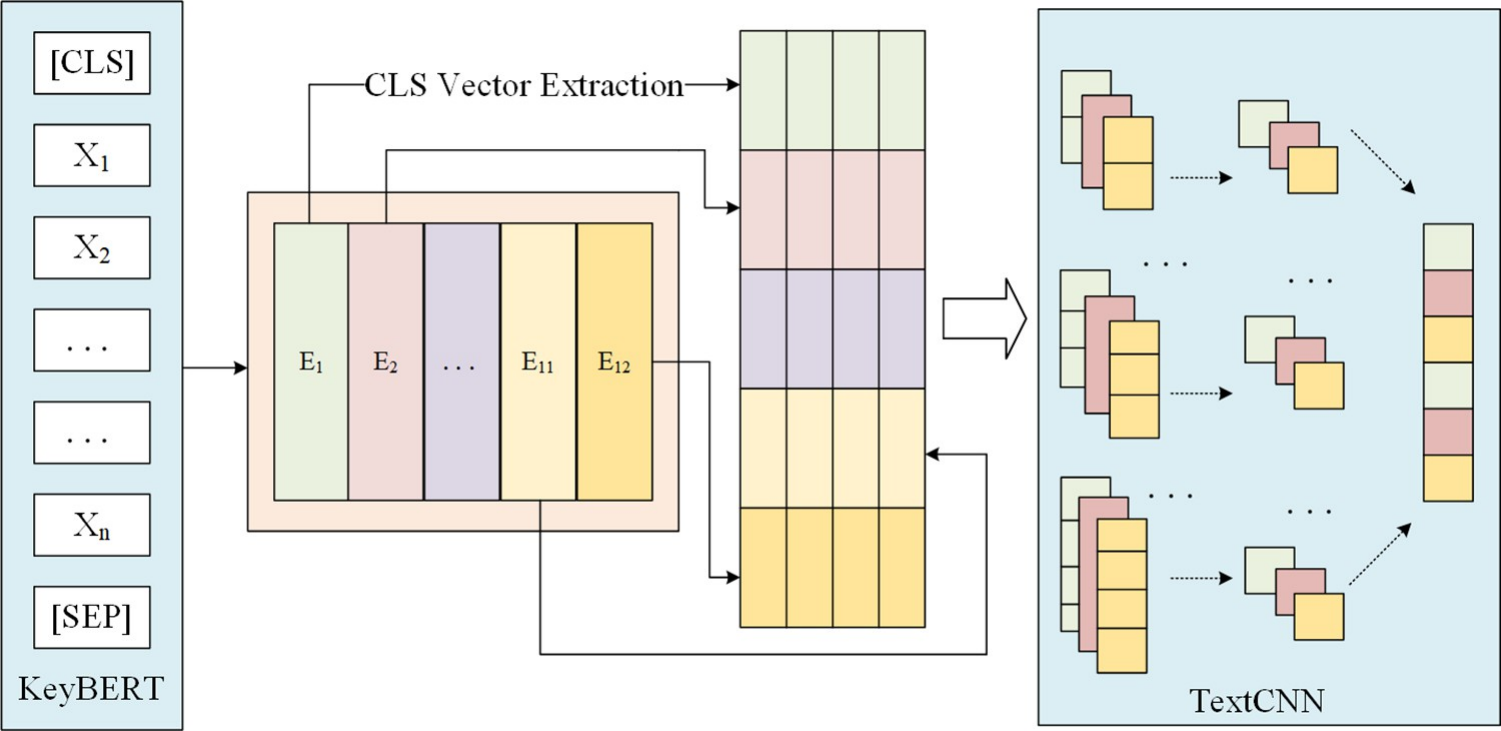
Topic classification based on KeyBERT-TextCNN.

After obtaining the topic keywords from the review texts, the next step is to perform label classification on these texts to identify the thematic categories to which the reviews belong. TextCNN utilizes convolutional layers to extract features from text and achieves text classification through pooling layers and fully connected layers [44]. The specific network architecture is illustrated in Fig 3.

The SnowNLP library offers a pre-trained sentiment analysis model that has been trained on a large-scale Chinese corpus. SnowNLP employs a comprehensive sentiment analysis approach by calculating the sentiment score of each word in the text while considering semantic and contextual information [45]. This method enables SnowNLP to determine not only the overall sentiment polarity of the text but also to provide specific sentiment scores.

### 3.2 Perceived risk prediction model

#### 3.2.1 Label generation based on PCA-K-medoids clustering

In risk prediction, obtaining high-quality sample labels is crucial for model training. However, manual labeling of samples is not only time-consuming and subjective, but it may also introduce labeling bias, which can further affect model performance. To address this issue, this study proposes an automated sample labeling generation method based on the PCA-K-medoids clustering algorithm. PCA projects high-dimensional data into a lower-dimensional space through linear transformation, while retaining as much of the data’s informational content as possible, which helps solve issues of correlation and redundancy between indicators [46]. K-medoids clusters data by selecting actual sample points as cluster centers, offering robust performance and high interpretability [47]. The training process is presented in Table 3.

**Table 3:** Pseudocode for the PCA-K-medoids clustering algorithm.

**Algorithm**: PCA-**K-medoids**
**Input**: Sample set X’ = {x₁, x₂,. . ., xₙ}, Number of clusters K, Desired explained variance ratio r
**Output**: K clusters and their medoids, Silhouette coefficient S
1 Perform PCA on X’ to reduce dimensionality: X = PCA(X’, explained_variance_ratio = r)
2 Randomly select K initial medoids C = {c₁, c₂,. . ., cₖ} from X
3 for each x_i_ ∈ X do
4 Calculate distance d(x_i_, cⱼ) from x_i_ to each medoid cⱼ
5 Assign x_i_ to cluster j where j = argmin d(x_i_, cⱼ)
6 end for
7 for each cluster j do
8 Find new medoid that minimizes sum of absolute errors:
cⱼ_new = argmin ∑ d(x_i_, x), x_i_ ∈ cluster j
9 end for
10 if all cⱼ_new = = cⱼ then
11 break
12 else
13 Update medoids: C = {c₁_new, c₂_new,. . ., cₖ_new}
14 end if
15 until maximum iterations reached or convergence
16 Calculate Silhouette coefficient S:
S = (1/n) ∑_i_ (b(i)—a(i)) / max{a(i), b(i)}
where a(i) is the average distance between i and points in its cluster
b(i) is the average distance between i and points in the nearest other cluster
17 Return K clusters, medoids, and Silhouette coefficient S

#### 3.2.2 Perceived risk prediction using XGBoost algorithm

The core principle of XGBoost is to iteratively build a series of decision trees to minimize the loss function. Each new decision tree is trained on the residuals of the previous tree, thereby progressively approximating the optimal solution [48]. During the model training process, we employed grid search and cross-validation techniques to optimize the hyperparameters of XGBoost, including the number of trees, tree depth, and learning rate, thereby enhancing the model’s performance and generalization capability. The mathematical model is represented by Eqs (1) and (2) as follows:

$${\hat{y}}_{i}^{\left(t\right)}=\sum_{k=1}^{t}f_{k}(x_{i})={\hat{y}}_{i}^{\left(i-1\right)}+f_{t}(x_{i}),f_{t}\in F$$ (1)

$$F=f(x_{i})=w_{q(x)}$$ (2)

In Eqs (1) and (2), *i*∈(1,2,…,*n*) represents the sample size, where n is the total number of samples. ${\hat{y}}_{i}$ denotes the predicted value of the model, and *x_i_* represents the perceived risk evaluation indicator. The variable *t* signifies the number of sub-models. *w_q(x)_* is the weight vector of all leaf nodes in the XGBoost model. *f_k_* represents the weight of the leaf node in the k-th regression tree, and *F* denotes the ensemble of all regression trees.

## 4 Results and discussion

### 4.1 Data sources and preprocessing

JD.com (JD) is one of the most popular Business-to-Consumer (B2C) e-commerce platforms in China. To evaluate the comprehensive service characteristics of merchants, JD assigns an overall score to each merchant based on their recent transaction records. These characteristics encompass various aspects, including transaction disputes, logistics fulfillment, after-sales service, customer service consultation, and store ratings. Given the diversity of product types on the platform and the need to ensure data representativeness, this study has selected Bluetooth headphones, mobile phones, air conditioners, and facial creams as research subjects, based on the following representational considerations: these four categories cover a wide price range from high-end to entry-level, reflecting the purchasing behaviors of different consumer groups; they are widely used in modern life, representing the daily needs and preferences of most consumers; in brand diversity, they include both international and local brands, adding a degree of randomness.

For each category of products, we employed web scraping technology to collect online consumer reviews, product details, and seller characteristic data from the top 1,000 merchants ranked by overall performance. To ensure the validity of the reviews, comments with fewer than 5 characters and invalid reviews were removed. In terms of product information, price and sales volume were extracted as key features. Missing values in merchant characteristics were filled using the mode. After data cleaning and preprocessing, the final dataset includes product information and merchant characteristics from 3474 stores, along with 262,752 online reviews. The relevant data mentioned in the manuscript can be found in S1 Table. Crawling technology and analytical methods comply with the terms and conditions of the data source.

### 4.2 Analysis results of online review topic features

#### 4.2.1 Results of topic feature extraction from online reviews

In this study, BERT Embeddings were generated using the Sentence-Transformers package, with the distilbert-base-nli-mean-tokens model selected as the pre-trained model. The model parameters were configured as follows: MMR (Maximal Marginal Relevance) was set to 0.15, N-Gram range was (1,1), and Top_n was set to 15. All other parameters were left at their default values. Using the KeyBERT algorithm, this study identified 13 representative topics, which were subsequently categorized into four main dimensions of perceived risk: functional risk, quality risk, service risk, and appearance risk. The results are presented in Table 4. The results of the thematic feature extraction of online reviews for other products can be found in S2 Table.

**Table 4:** Topic clustering and dimensional classification of online reviews. A Case Study of Bluetooth Headphones.

Perceived Risk Dimensions Clustering	Topic Code	Supporting Text Count	Top 8 Feature Words Belonging to Each Topic
Functionality	T1	9742	Sound, Sound Quality, Timbre, Noise, Treble, Recording, Sound Leakage, Bass
	T2	3486	Matching, Adaptation, Pairing, Combination, Capability, Performance, Stability, Running
	T3	3346	Latency, Voice communication, Wireless, Bluetooth, Connection, Signal, Disconnection, Intermittent
Quality	T4	8093	Quality, Craftsmanship, Tactile sensation, Product quality, Headphones, Earbuds, Secondary headphones, Weight
	T5	4231	Authentic product, Price-performance ratio, Cost-effective, High quality at a low price, Exceeding value expectations, Promotional offer, Price
	T6	3334	Second-hand, Product, Item, Counterfeit, Refurbished, Damage, Injure, Durability
	T7	4156	Battery life, Standby time, Lifespan, Battery, Battery level, Charging, Fully charged, Current noise
Service	T8	4755	Service, Customer service, Online shopping, Merchant, Retailer, Enthusiasm, Attentiveness, Attitude
	T9	3896	After-sales service, Return, Logistics, Dispatch, Exchange, Transportation, Distribution
	T10	4035	Review, Description, Seller, Express delivery, Guarantee, Five-star rating, Price reduction, Price protection
Appearance	T11	3036	Headphone case, Charging case, Charging dock, Outer shell, Protective cover, Case, Box, Lid
	T12	2085	Appearance, Design, Aesthetic appeal, Dimensions, Size, Compact, Exquisite, Elegant
	T13	2086	Packaging, Scratch, Scrape, Wear mark, Trace, Dust, Stain, Gap

In terms of functional risk, consumers are primarily concerned about the sound quality of headphones (T1), including audio fidelity and background noise. Following that, the fit and stability of the headphones (T2), as well as Bluetooth connectivity and call experience (T3), are also important. This indicates that sound quality and connection stability are the main factors consumers consider when evaluating functional risk. Under the quality risk dimension, consumers pay significant attention to the overall quality and craftsmanship of the headphones (T4), reflecting that product quality and perceived value play a crucial role in their perception of quality risk. Regarding service risk, consumers place considerable importance on the service quality (T8) and after-sales support (T9) provided by suppliers, including return and exchange policies and logistics efficiency. Additionally, consumers are attentive to the reputation and word-of-mouth of the suppliers (T10), which shows that the credibility of suppliers also affects consumers’ perception of risk.

#### 4.2.2 Sentiment analysis based on topic classification results

A rule-based approach utilizing keyword matching was employed to generate a labeled dataset comprising 103,545 entries. Subsequently, a TextCNN model was trained on this annotated corpus of review data. The model architecture comprises a 768-dimensional word embedding layer, multiple convolutional layers, a global max pooling layer, and two fully connected layers. The model was trained using sparse categorical cross-entropy as the loss function, Adam optimizer, and accuracy as the evaluation metric. Four distinct parameter configurations were selected for experimental evaluation. The specific parameter settings are presented in Table 5.

**Table 5:** Parameter configuration for TextCNN model.

Index	Kernel Size	Number of Kernels	Dropout
Configuration 1 (Baseline)	[3, 4, 5]	256	0.5
Configuration 2 (Increased Kernel Count)	[3, 4, 5]	384	0.5
Configuration 3 (Adjusted Kernel Sizes)	[2, 3, 4, 5, 6]	256	0.5
Configuration 4 (Modified Dropout)	[3, 4, 5]	384	0.3

**Fig 4:**
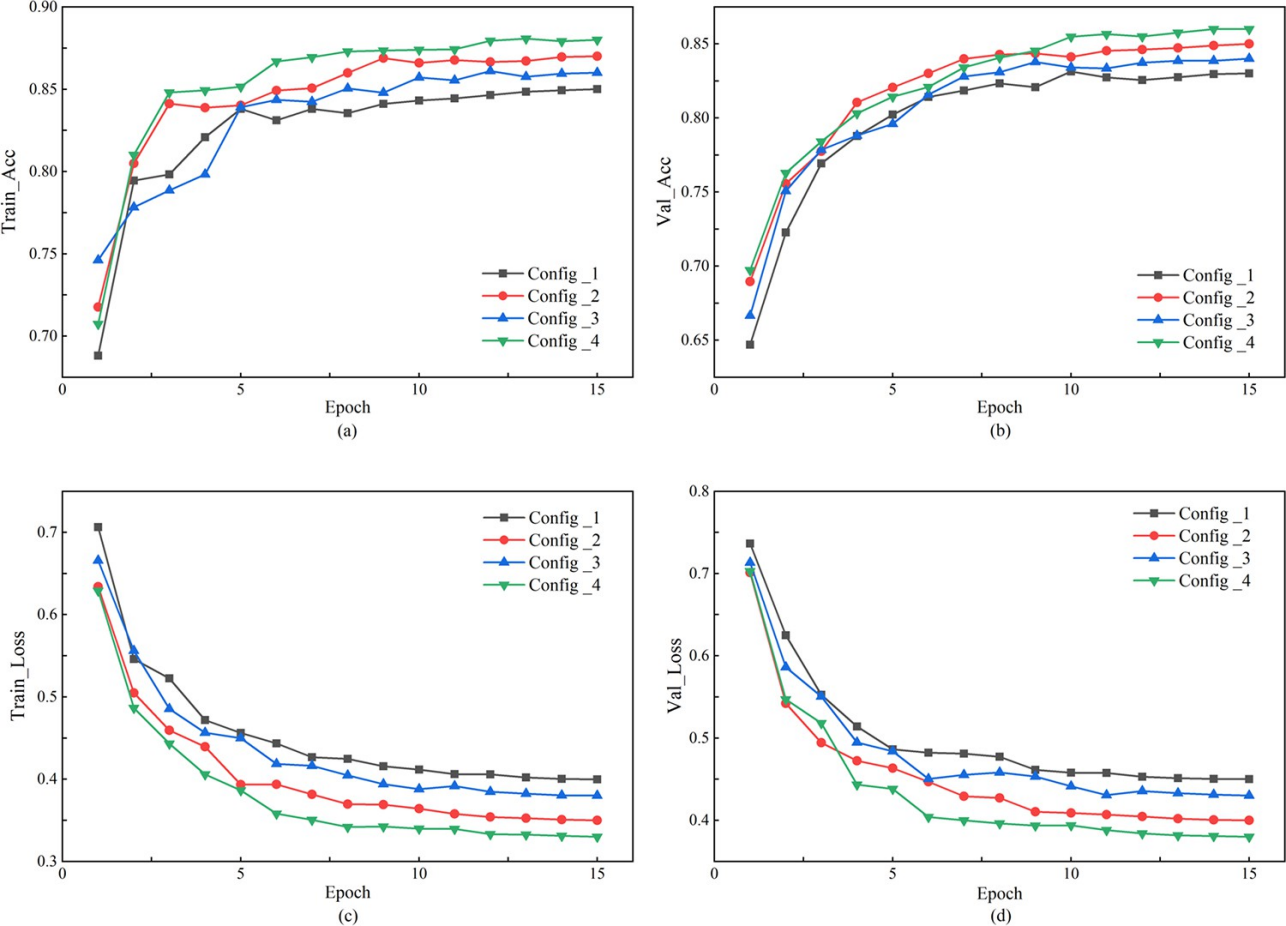
Model performance on the validation set.

The model’s performance on the validation set is illustrated in Fig 4, where one epoch is defined as a complete pass through the entire sample set. The TextCNN model based on Configuration 4 exhibited superior performance, demonstrating high accuracy and low loss on the validation set. This configuration effectively balances the model’s generalization capability while mitigating overfitting. Consequently, this optimized model was selected for the thematic classification of all review texts in the corpus.

**Fig 5:**
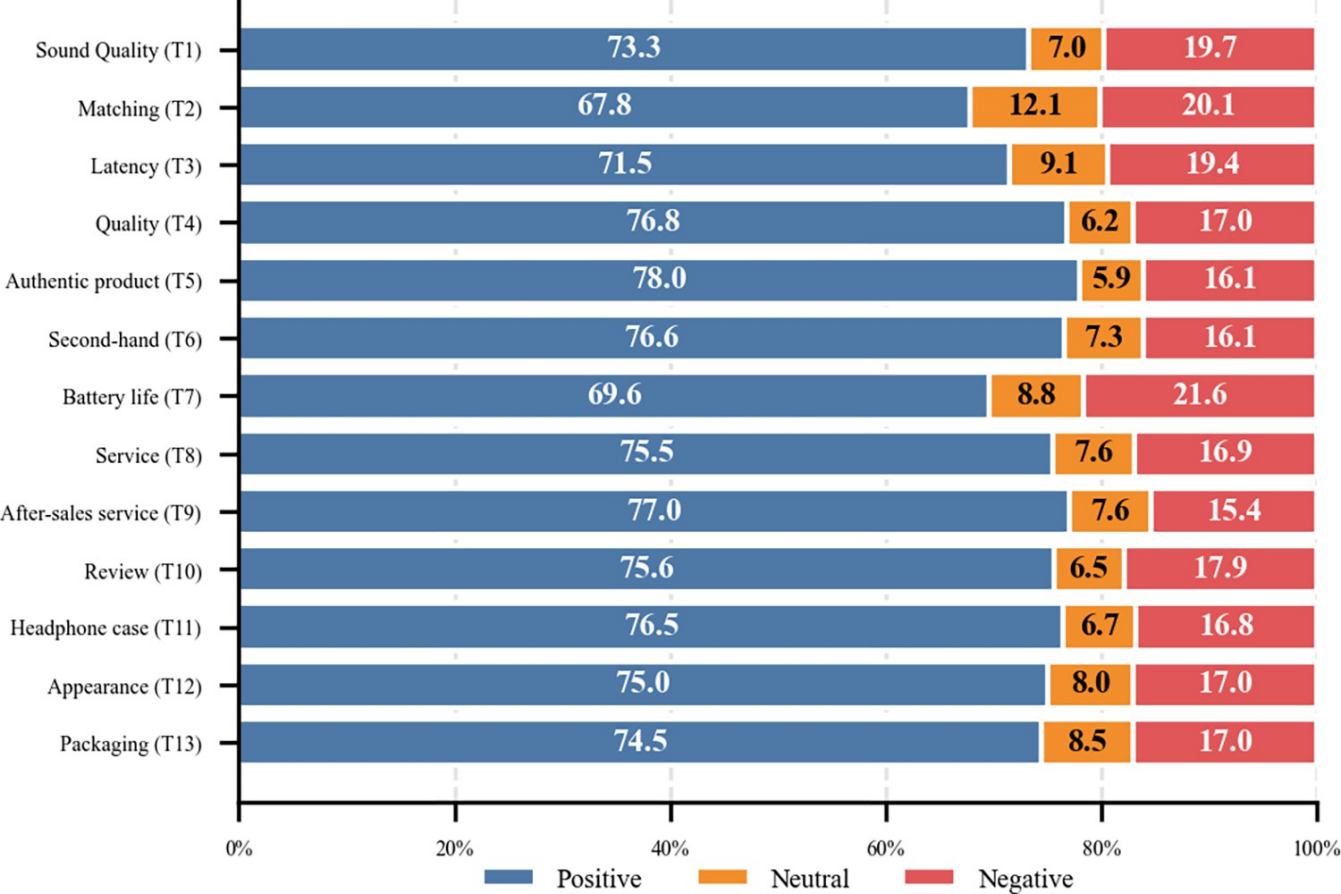
Topic-based sentiment analysis: A case study of Bluetooth headphones.

Based on the classification results of the model, the sentiment of the review text is quantified, and for each shop, the sentiment intensity of all its review data is calculated using the Snownlp library. The formula is $\sum_{i=1}^{n}\frac{sent_{i}}{n}$, where *sent_i_* is the sentiment polarity of the i-th comment, with positive sentiment tending towards 1 and negative sentiment tending towards 0, and n is the number of comments. The sentiment analysis results in Fig 5 show that T7, T2, and T1 have the highest proportion of negative emotions, which belong to the quality dimension and the functionality dimension, respectively.

### 4.3 Sample clustering results

**Fig 6:**
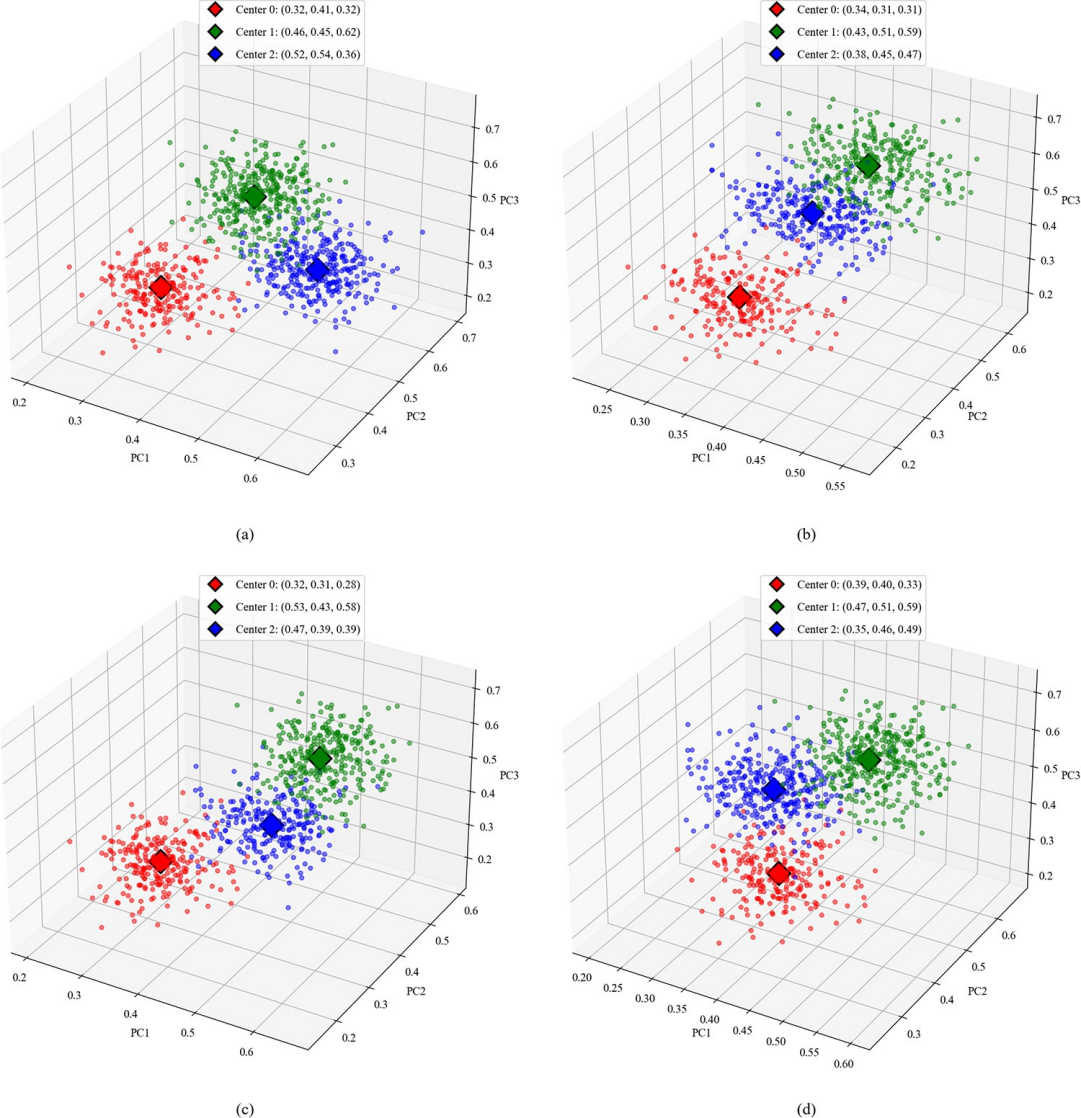
Clustering results based on PCA-K-medoids algorithm. (a)-(d) represent the perceived risk clustering results for headphones, mobile phones, air conditioners, and facial cream, respectively.

Before clustering using PCA-K-medoids, it is necessary to standardize the indicators for easier comparison. In this study, we adopted a reverse transformation method, where higher indicator values correspond to lower risk levels. The visualization of the clustering results is presented in Fig 6.

### 4.4 Risk prediction results of XGBoost

**Fig 7:**
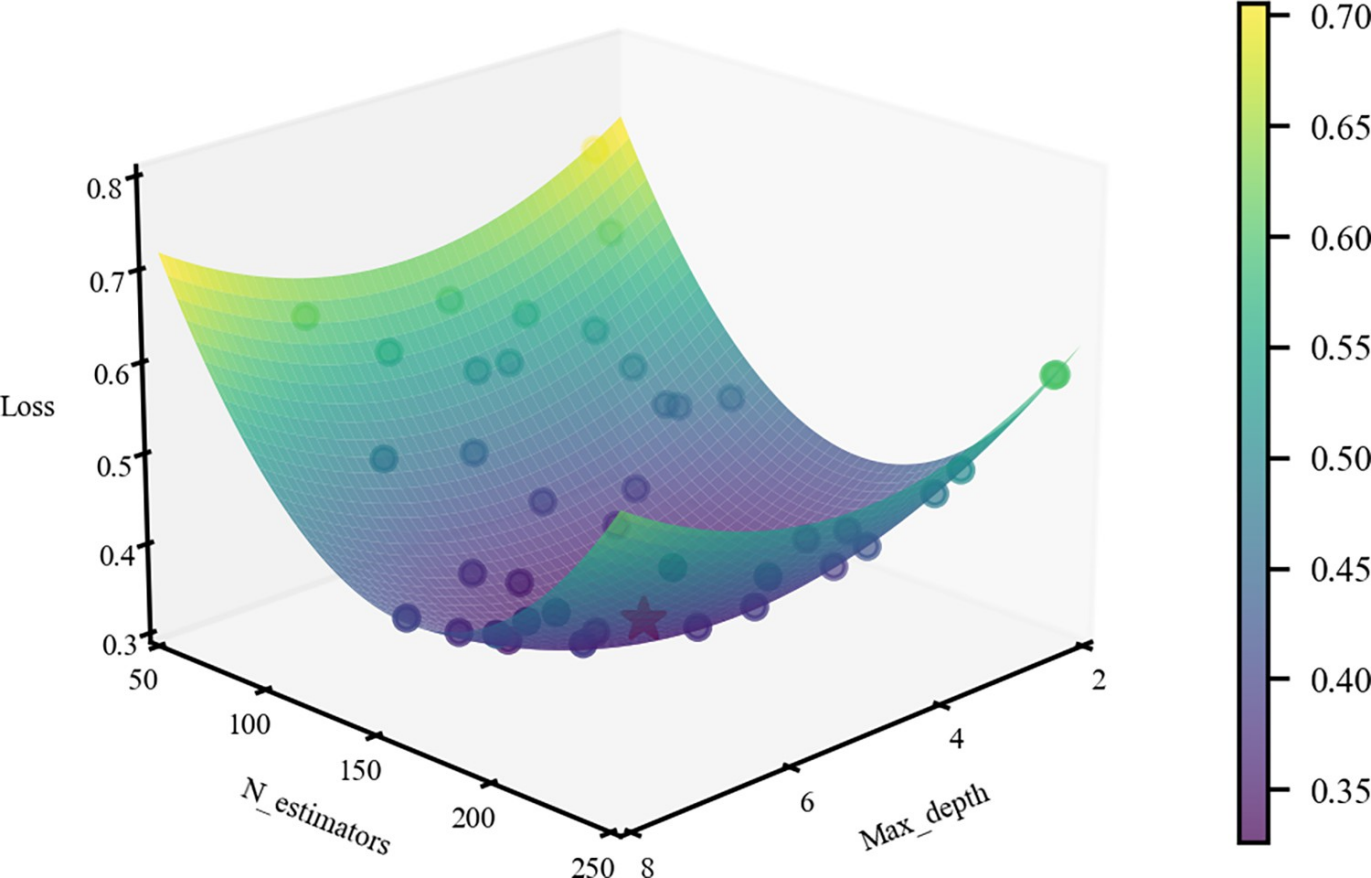
Grid search process.

This article uses multi-class logarithmic loss (mlogloss) as the optimization objective and employs grid search (GR) for hyperparameter tuning. The dataset is divided into a training set and a test set in an 8:2 ratio. We set the learning rate to 0.1, subsample to 0.8, and colsample_bytree to 0.8. This article conducts an in-depth validation of two important parameters for XGBoost: Max_depth and N_estimators, as illustrated in Fig 7. As the grid search progresses, the value of the loss function continues to decrease. The optimal parameter combination yields a Loss of 0.32, with Max_depth = 5 and N_estimators = 160.

The model’s performance on the test set, including precision, recall, and F1 score, is presented in Table 6. The prediction results indicate that the model achieved an accuracy of 0.87, suggesting a relatively precise prediction of overall perceived risk in online shopping. This demonstrates the model’s effectiveness in distinguishing between different cluster types.

**Table 6:** Performance evaluation of models on test dataset.

True Labels	Precision	Recall	F1 Score
Class 0	0.82	0.75	0.78
Class 1	0.79	0.94	0.86
Class 2	0.86	0.88	0.87
Accuracy	0.87		
Macro Average	0.83	0.86	0.84
Weighted Average	**0.84**	**0.86**	**0.85**

**Fig 8:**
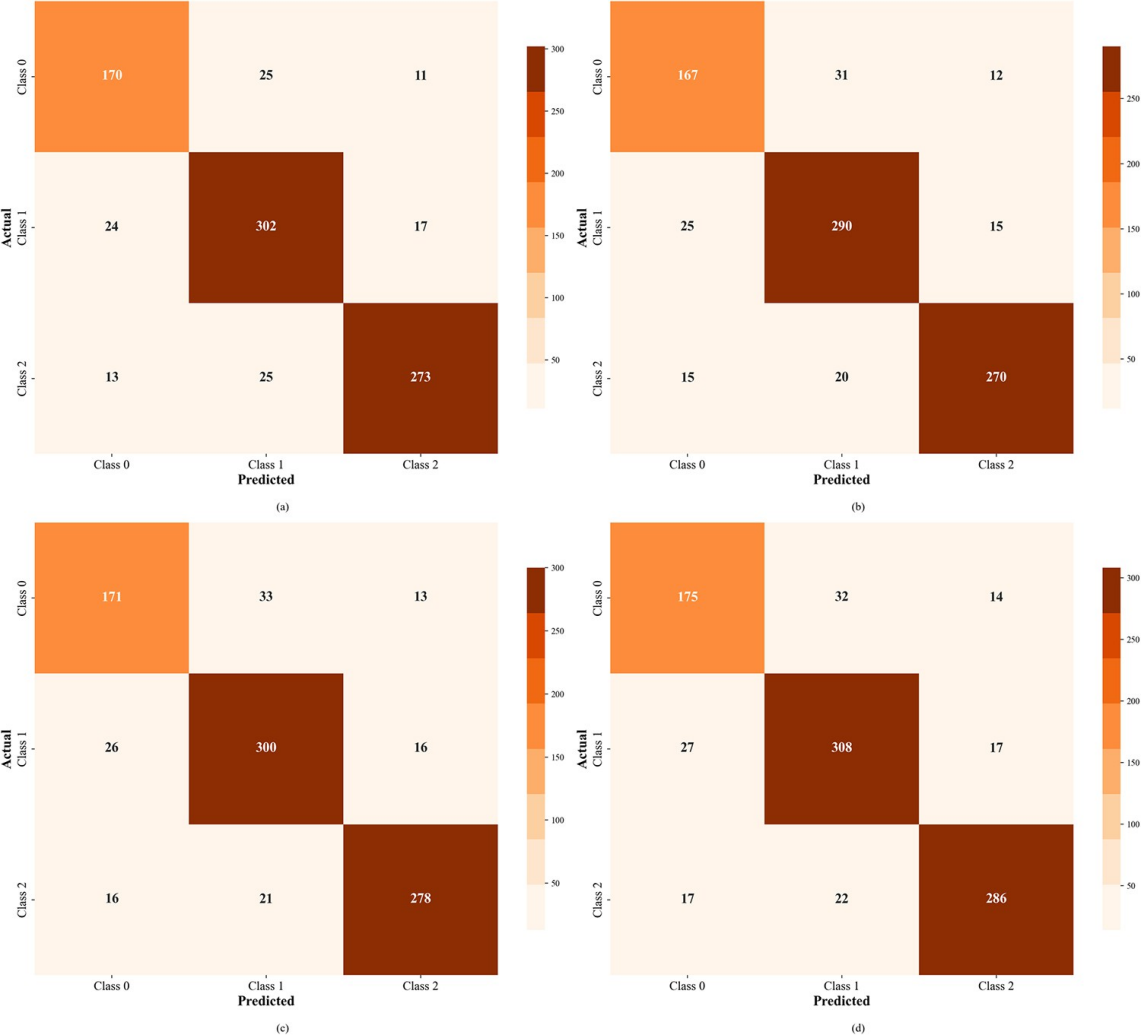
Confusion matrix. (a)-(d) represent the perceived risk prediction results for headphones, mobile phones, air conditioners, and facial cream, respectively.

In order to explore the misclassification of risk types, this paper utilizes a confusion matrix visualization, as shown in Fig 8. Clearly, the model exhibits high accuracy along the diagonal, with particularly good performance for categories 1 and 2. However, there are some challenges in classifying category 0, which indicates issues such as data imbalance and insufficient feature extraction in the dataset.

#### 4.4.1 Comparative analysis of models

To further assess the performance of the models, this paper selects several representative models and benchmark models for comparison. The parameter settings for each model are as follows: for the BSVM, a Gaussian kernel function is used, with the penalty coefficient C set to 10 and the kernel parameter gamma set to 0.1. The prior probability distribution is defined as a Gaussian distribution. LightGBM uses default parameters, with a subsampling ratio of 0.8, a feature sampling ratio of 0.8, and an early stopping round set to 100. The CNN architecture consists of three convolutional layers and two fully connected layers, with the number of convolutional kernels set to 32, 64, and 128, respectively, while max pooling is applied in the pooling layers. The benchmark models mainly include logistic regression(LR), decision trees(DT), random forests(RF), and K-nearest neighbors (KNN). The parameter settings for these benchmark models mainly adopt default values but have been appropriately adjusted based on the characteristics of the multi-classification tasks.

The predictive performance of each model is shown in Table 7. As indicated in the table, the risk prediction model based on GR-XGBoost demonstrates more stable performance and higher accuracy, consistently outperforming those that use other algorithms.

**Table 7:** Performance comparison of different models.

Model	Accuracy	Precision	Reacall	F1-score
GR-XGBoost	**0.87**	**0.84**	**0.86**	**0.85**
LightGBM	0.72	0.68	0.72	0.7
CNN	0.69	0.73	0.71	0.72
RF	0.68	0.66	0.68	0.67
BSVM	0.65	0.63	0.67	0.65
KNN	0.63	0.61	0.63	0.62
DT	0.61	0.59	0.61	0.6
LR	0.58	0.56	0.58	0.57

#### 4.4.2 Model interpretability analysis

**Fig 9:**
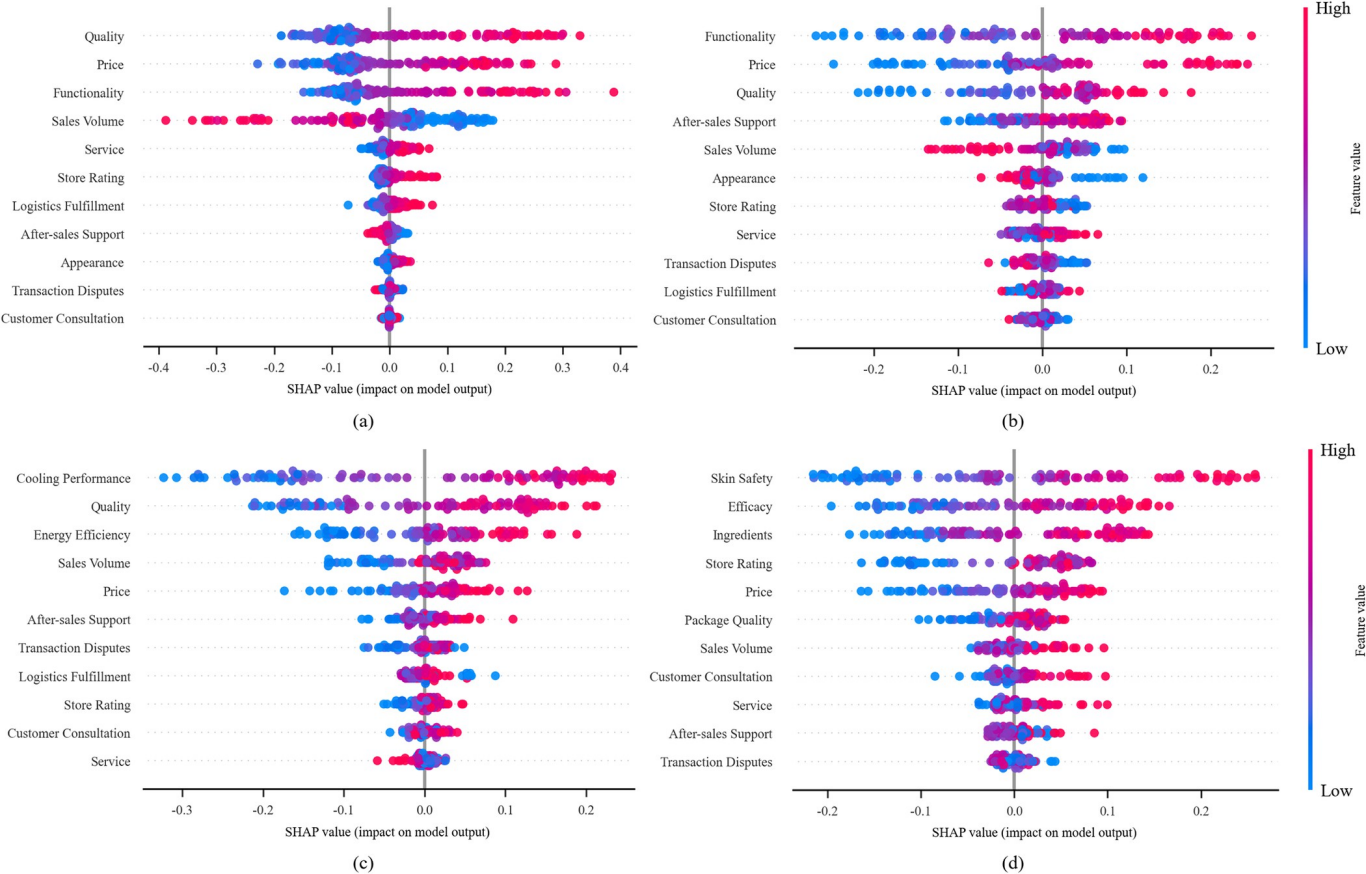
Explainability analysis. The y-axis represents various features sorted by their importance; the x-axis indicates SHAP values, with the positive direction representing normal clustering and the negative direction indicating high-risk clustering. Each point represents a sample in the dataset, with high values (red) corresponding to positive influence and low values (blue) indicating negative influence. (a)-(d) represent the interpretability analysis of perceived risk prediction results for headphones, mobile phones, air conditioners, and facial cream, respectively.

To further identify the key factors influencing consumers’ overall perceived risk, we adjusted the clustering labels, combining Class 1 and Class 2 as the normal perceived risk cluster for comparison with the high perceived risk cluster of Class 0. SHAP was integrated into the output layer of the XGBoost model. SHAP assigns a Shapley value to each feature and simulates different orders of feature inclusion to measure their contribution to the model output [49]. Fig 9 presents the model interpretability results based on SHAP values.

Fig 9 indicates that perceived risk exhibits significant heterogeneity across different product categories. In subfigures (a) and (b), the effects of quality, price, and functional features on the model output are the most pronounced, with a wide distribution of SHAP values. This suggests that improving product quality and highlighting product features are key factors in reducing perceived risk for consumers. Our research reveals an intriguing inverse relationship between price points across different product categories and perceived risk. Specifically, when consumers encounter products positioned at a high price point, their perceived risk significantly decreases. This relationship indicates that consumers use price as a heuristic tool to reduce risk, with higher prices serving as a quality assurance mechanism, which aligns with the price-quality inference theory proposed by Guizzardi et al [50]. Furthermore, this inverse relationship is moderated by the characteristics of product categories. In high-involvement products, such as electronics, where functional risk is greater, this effect is more pronounced, while in experiential goods, such as creams, other attributes like safety and efficacy have a stronger influence on risk perception. Sales exhibit a clear bimodal distribution, where both low and high sales volumes may increase perceived risk, while moderate sales volumes tend to reduce perceived risk. This may reflect the dual influence of the “scarcity effect” and the “herding effect” [51]. It is noteworthy that service-related characteristics, such as customer inquiries and transaction disputes, consistently show relatively low importance across all product categories. Although certain specific product attributes dominate perceived risk (for example, the cooling performance of air conditioners and the skin safety of creams), some common factors like quality and price remain significantly important across multiple product categories [52, 53].

## 5 Conclusions and limitations

This study constructs a fine-grained online shopping perceived risk prediction model based on multi-dimensional feature fusion, which possesses strong interpretability and practical guidance significance. Empirical research results indicate that this model can provide data-driven decision support to reduce users’ perceived risks and optimize merchants’ risk management strategies. For product developers and marketing practitioners, the research findings emphasize the significant impact of intrinsic product attributes on perceived risk. Recommendations include: (1) prioritizing product quality and functionality optimization as core strategies to reduce users’ perceived risks; (2) continuously improving product functionality indicators and highlighting differentiated features to meet consumers’ heterogeneous needs; (3) conducting precise market segmentation and targeting customer positioning based on sales characteristic data.

Practical recommendations for e-commerce platforms include: (1) optimizing the product information display system by providing standardized product specifications and high-resolution multimedia content to reduce information asymmetry; (2) developing user experience-based feature comparison tools to lessen the cognitive burden on consumers during the product evaluation process; (3) implementing a transparent price protection mechanism that enhances the perception of price fairness through visualized price fluctuation data, thereby boosting consumer purchasing confidence.

Despite these findings, the current model has several limitations, including issues related to data representativeness and the comprehensiveness of feature selection. Future research should further expand the data dimensions by incorporating consumer purchase behavior data, product images, videos, and other unstructured data to dynamically characterize perceived risk from multidimensional perspectives.

## Supporting information

S1 Table Relevant data underlying the findings described in manuscript. (XLSX)

S2 Table Topic clustering and dimensional classification of online reviews. (XLSX)
